# Long-Term Outcomes of Simultaneous Integrated Boost in Node-Positive Cervical Cancer: A Single-Institution Retrospective Study

**DOI:** 10.7759/cureus.94828

**Published:** 2025-10-17

**Authors:** Sreenivasa Rao Bonala, Bala Venkat Subramanian, Cherwin Venati, Sorakayapet Arumugam Raja

**Affiliations:** 1 Radiation Oncology, Sri Venkateswara Institute of Medical Sciences, Tirupati, IND

**Keywords:** cervical cancer, intensity modulated radiotherapy, lymph node metastasis, simultaneous integrated boost, treatment outcomes, volumetric modulated arc therapy

## Abstract

Background

Nodal involvement in cervical cancer is associated with poor prognosis. Although modern radiotherapy techniques (intensity-modulated radiotherapy (IMRT)/volumetric-modulated arc therapy (VMAT)) enable safe nodal dose escalation, clinical data, particularly from Indian settings and node-positive subgroups, remain limited. This study evaluated outcomes and toxicity using a simultaneous integrated boost (SIB) to radiologically involved nodes.

Methods

We retrospectively analyzed 56 patients with node-positive, locally advanced cervical cancer treated between 2018 and 2023. All patients received pelvic external-beam radiotherapy (45-50 Gy) with nodal SIB (median, 55 Gy in 25 fractions), followed by intracavitary brachytherapy (24 Gy in 3 fractions or 28 Gy in 4 fractions). Weekly cisplatin was administered when feasible. Tumor and nodal responses were assessed at 3 months, and survival was estimated using Kaplan-Meier and compared with log-rank tests.

Results

After a median follow-up period of 35 months, complete response was observed in 46 (81.8%) and 51 (91%) patients with primary and nodal diseases, respectively. Initial failures were categorized as local in seven cases (12.5%), regional in six (10.7%), and distant in 15 (27.3%). The five-year local and regional control rates were 86% and 85%, respectively; the distant recurrence-free survival rate was 64%, and the overall survival rate was 68%. Patients with para-aortic nodal disease (IIIC2) exhibited inferior distant control compared to those with IIIC1 (median, 29 months vs. not reached; p = 0.047). Late grade ≥3 gastrointestinal and genitourinary toxicities were observed in two (3.6%) and one (1.8%) patients, respectively. Vaginal stenosis was reported in 14 (25.0%) patients, with grade 3 stenosis occurring in 2 (3.6%) patients.

Conclusions

Definitive chemoradiation with IMRT/VMAT using a simultaneous integrated nodal boost achieved high complete response rates, excellent pelvic control, and low rates of severe late toxicity in node-positive cervical cancer. Distant relapse remained the main pattern of failure, particularly in patients with para-aortic involvement. These findings support nodal dose escalation as a safe, practical strategy and highlight the need for systemic intensification in high-risk patients.

## Introduction

Carcinoma of the cervix ranks as the second most prevalent cancer among Indian women, with the majority of patients presenting with locally advanced cervical cancer (LACC) involving adjacent tissues or regional lymph nodes [1]. The standard therapeutic approach for LACC comprises pelvic external-beam radiotherapy (EBRT) in conjunction with weekly cisplatin, followed by intracavitary brachytherapy (ICBT) [2].

Lymph node (LN) involvement is recognized as an adverse prognostic indicator [3], and the 2018 FIGO staging revision now categorizes pelvic and para-aortic nodal diseases as stage IIIC1 and IIIC2, respectively [4]. Historically, randomized trials have established that pelvic EBRT at 45-50 Gy in 25 fractions, combined with concurrent chemotherapy and a brachytherapy (BT) boost, is adequate for disease without nodal involvement [5-7]. However, in cases where nodes are involved, higher doses are necessary for sustained control, which conventional radiotherapy (RT) cannot safely deliver beyond 50 Gy because of the risk of bowel toxicity [8,9].

Technological advancements, such as intensity-modulated radiotherapy (IMRT) and volumetric-modulated arc therapy (VMAT), now facilitate conformal dose escalation, allowing nodal boosting while sparing the surrounding normal tissues [10-12]. Several retrospective and prospective studies have demonstrated the feasibility of nodal dose escalation using IMRT or VMAT, with promising local control and acceptable toxicity profiles [13-22]. However, there is no uniform consensus regarding the optimal dose, technique, or target delineation, leading to heterogeneous global practices [23]. Data from Indian settings are particularly limited. Since 2018, our department has implemented a risk-adapted strategy utilizing IMRT/VMAT with a simultaneous integrated boost (SIB) for nodal disease in combination with concurrent cisplatin and BT [24,25]. Here, we present a retrospective study evaluating the outcomes and toxicity of this approach in patients with node-positive cervical carcinoma treated at our institution.

## Materials and methods

Study design and patient selection

After approval from the Institutional Review Board, we retrospectively reviewed the medical records of all patients with cervical carcinoma treated at the Department of Radiation Oncology, Sri Venkateswara Institute of Medical Sciences, Tirupati, between January 2018 and December 2023. During this period, 510 patients with cervical carcinoma underwent radiotherapy at our institution. Of these, 102 patients were excluded because they had received palliative radiotherapy or postoperative adjuvant treatment. Among the remaining 408 patients treated with radical intent, 80 had radiologically node-positive disease. Twenty-four patients were excluded from the analysis because they underwent sequential nodal boost with three-dimensional conformal radiotherapy (3D-CRT). The final study cohort comprised 56 patients with node-positive cervical carcinoma who were treated with definitive chemoradiation incorporating SIB. Data on patient demographics, tumor factors, treatment details, and outcomes were collected using a predesigned proforma by the principal investigator and were independently verified by two co-investigators.

All patients underwent a gynecological examination, and the diagnosis was confirmed histologically in all cases. Most patients underwent computed tomography (CT) or magnetic resonance imaging (MRI) of the abdomen and pelvis with chest X-ray for staging, while a subset underwent positron emission tomography-computer tomography (PET-CT). LN positivity was defined radiologically as nodes >10 mm in short-axis diameter or <10 mm with suspicious features, such as round shape, clustering, irregular margins, central necrosis, or contrast enhancement.

External-beam radiotherapy (EBRT) planning and delivery

All patients underwent CT simulation in the supine position using a vacuum-immobilization bag. Images were acquired from 2 cm above the diaphragm to the mid-thigh at a 3-mm slice thickness, with a comfortably full bladder and empty rectum. EBRT was delivered using IMRT or VMAT at a dose of 45 Gy in 25 fractions to the primary tumor and elective nodal regions, with an SIB of 55 Gy in 25 fractions to radiologically abnormal nodes.

Clinical target volumes (CTV) included both primary and nodal disease, and organs at risk (OARs: rectum, bladder, bowel bag, and sigmoid) were delineated according to international guidelines [26-28]. The primary CTV encompassed the gross cervical tumor, cervix, uterus, parametrium, adnexa, and proximal vagina (3 cm below the gross disease or the entire vagina if involved). The nodal CTV included the internal, external, common iliac, obturator, and presacral nodes. For pelvic node-positive cases, elective para-aortic nodes were contoured up to the infrarenal vessels; in patients with para-aortic involvement, the contour was extended up to the lower border of T10.

The planning target volume (PTV) was generated by adding 1.5-2 cm margins to the primary CTV and 7-10 mm to the nodal CTV. The combined elective PTV (PTV45) was prescribed to a dose of 45 Gy. The GTV node was expanded by 5 mm to generate the CTV nodal boost (edited from anatomical barriers), and an additional 5 mm margin was added to derive the PTV nodal boost.

The OAR constraints included a bowel bag V45 <200 cc, bilateral kidney mean dose <15 Gy, and femoral head Dmax <50 Gy. PTV coverage was prioritized over bladder and rectal-sparing. All patients were treated in the supine position with bladder filling and rectal emptying protocol. The setup was verified using cone-beam CT. EBRT was delivered using an Elekta Synergy linear accelerator (Elekta AB, Stockholm, Sweden). Patients eligible for chemotherapy received concurrent weekly cisplatin chemotherapy (40mg/m2).

Brachytherapy

Following EBRT, patients received ICBT, delivering 24-28 Gy using either 8 Gy × 3 fractions or 7 Gy × 4 fractions, administered once or twice weekly for 2-4 weeks. According to the institutional protocol, BT was interdigitated with EBRT in most cases. Fresh CT-based contouring and individualized planning were performed for each fraction. Both point A-based and volume-based plans were employed. The high-risk clinical target volume (HR-CTV) was delineated according to the guidelines [24]. The cumulative prescribed dose corresponded to an equivalent dose in 2 Gy fractions (EQD2) of approximately 85 Gy10 and 75 Gy10 to the dose received by 90% (D90) and 98% (D98) of the HR-CTV, respectively, while respecting OAR constraints of ≤75 Gy3 for the rectum and sigmoid and ≤90 Gy3 for the bladder, consistent with GEC-ESTRO recommendations [24]. All treatments were delivered using an Iridium-192 source with a high-dose-rate remote afterloading system (GammaMed Plus IX, Varian).

Follow-up and outcome assessment

Post-treatment, patients were followed up every three months for the first two years and every six months thereafter. Tumor response was assessed at three months post-treatment, both clinically and radiologically (CT/MRI/PET-CT as appropriate), according to the RECIST criteria [29]. Late toxicities were measured from 90 days after completion of RT according to the Radiation Therapy Oncology Group (RTOG) criteria [30].

Statistical analysis

Descriptive statistics were used to summarize the patient, tumor, and treatment characteristics. Categorical variables are expressed as frequencies and percentages, and continuous variables as mean ± standard deviation or median with range, as appropriate.

Overall survival (OS), calculated from the date of the first fraction of EBRT to the date of death. Local recurrence-free survival (LRFS), regional recurrence-free survival (RRFS), and distant recurrence-free survival (DRFS) were calculated from the date of the first fraction of EBRT to the event (local/regional/distant recurrence). Survival curves were generated using the Kaplan-Meier method, and differences in survival between subgroups (e.g., FIGO stage IIIC1 vs. IIIC2) were compared using the log-rank test. Analyses were conducted with IBM SPSS Statistics v25.0 (IBM Corp., Armonk, NY, USA). A two-sided p < 0.05 was considered statistically significant.

## Results

Patient, tumor, and treatment characteristics

A total of 56 patients with node-positive, locally advanced cervical carcinoma were included in this study. The mean age at diagnosis was 54.0 years (range, 33-74 years), and the mean pretreatment hemoglobin level was 10.4 g/dL (range, 5.0-15.0 g/dL), with eight (14.3%) patients presenting with hemoglobin levels ≤8 g/dL. Comorbidities were present in 17 (30.4%) patients, most commonly hypertension and diabetes, and five (8.9%) were HIV-positive. Hydroureteronephrosis was observed in 16 (28.6%). Histologically, squamous cell carcinoma was observed in 52 (92.8%) patients, followed by adenocarcinoma in three (5.4%) and neuroendocrine carcinoma in one (1.8%). The median primary tumor width was 5.3 cm (range, 1.3-11.3 cm), and the median tumor volume was 102.3 cc (range, 2-400 cc). Pelvic nodal involvement (stage IIIC1) was present in 42 (75.0%) patients, whereas both pelvic and para-aortic nodal diseases (stage IIIC2) were observed in 14 (25.0%) patients. The detailed baseline and treatment characteristics are summarized in Table 1.

**Table 1:** Baseline, Tumor, and Treatment Characteristics of Patients (N = 56) Baseline demographics, tumor factors, and treatment details of the cohort. Values are presented as n (%) or mean/median (range). Descriptive statistics only; no comparative tests performed. N, n = Number of patients; HDUN: Hydroureteronephrosis; SCC: squamous cell carcinoma; LN: lymph node; U/L: unilateral; B/L: bilateral; EBRT: external-beam radiotherapy; IMRT: intensity-modulated radiotherapy; VMAT: volumetric-modulated arc therapy; BT: brachytherapy

Characteristic	Values
Age (years); mean (range)	54.0 (33–74)
Hemoglobin (g/dL); mean (range)	10.4 (5.0–15.0)
Comorbidities, n (%)	
No comorbidities	38 (67.9%)
With comorbidities	17 (30.4%)
HIV reactive	5 (8.9%)
HDUN; n (%)	
Yes	16 (28.6%)
No	40 (71.4%)
Histology; n (%)	
SCC	52 (92.8%)
Adenocarcinoma	3 (5.4%)
Neuroendocrine	1 (1.8%)
Tumor size (cm); median width (range)	5.3 (1.3–11.3)
Tumor volume (cc); median (range)	102.3 (2.0–400.0)
LN status; n (%)	
Pelvic LN only (IIIC1)	42 (75.0%)
Pelvic + Para-aortic LN (IIIC2)	14 (25.0%)
Median size of largest LN, cm (range)	2.7 (1.0–8.0)
Median number of LN per patient (range)	3 (1–8)
Parametrial Involvement	
No	10 (17.8%)
Medial (U/L or B/L)	29 (51.8%)
Lateral (U/L or B/L)	17 (30.4%)
EBRT dose (Gy); median (range)	55 (54–60)
Technique; n (%)	
IMRT	26 (46.4%)
VMAT	30 (53.6%)
BT regimen; n (%)	
28 Gy/4 fractions (7 Gy × 4)	39 (70%)
24 Gy/3 fractions (8 Gy × 3)	14 (25%)
Incomplete BT; n (%)	3 (5.4%)
Concurrent chemotherapy (cisplatin) received; n (%)	
Yes	40 (71.4%)
No	16 (28.6%)
Number of cycles of cisplatin; n (%)	
<4 cycles	23 (57.5%)
≥4 cycles	17 (42.5%)
Cumulative cisplatin dose; n (%)	
<200 mg/m²	7 (18.9%)
≥200 mg/m²	30 (81.1%)

Tumor and nodal response and late toxicities

At three months post-treatment, a complete response was achieved in 46 (81.8%) and 51 (91.1%) patients at the primary site and lymph nodes, respectively, whereas a partial response occurred in eight (14.5%) and five (8.9%) patients at the primary site and lymph nodes, respectively. The first recurrence involved the cervix in seven (12.5%), regional nodes in six (10.7%), and distant metastases in 15 (27.3%) patients. The median time to recurrence was 15 months for local (range, 12.1-17.3), 16 months for regional (range, 9.6-24.7), and 15.5 months for distant (range, 4.6-29) recurrences. All five HIV-positive patients were receiving highly active antiretroviral therapy (HAART) and demonstrated good response to cancer treatment, with no recurrences reported during follow-up. Among these, two patients died after two years due to non-cancer-related causes (breathlessness secondary to lung infection), as per institutional Antiretroviral Therapy (ART) center records. Overall, treatment was well tolerated across the cohort. Grade ≥3 late gastrointestinal toxicity occurred in two (3.6%) patients, and grade ≥3 genitourinary toxicity occurred in one (1.8%) patient. Vaginal stenosis occurred in 14 (25.0%) patients, including 2 (3.6%) with grade 3 stenosis. The findings are presented in Table 2.

**Table 2:** Tumor Response, Recurrence Patterns, and Late Toxicities (N = 56) Response assessment at three months and late toxicities during follow-up. Values are presented as n (%) and median (range) where applicable. Descriptive statistics only; no comparative tests performed. N: Number of patients; GI: Gastrointestinal; GU: Genitourinary

Outcome	n (%)
Primary Tumor Response	
Complete Response (CR)	46 (81.8%)
Partial Response (PR)	8 (14.5%)
Stable/Progressive Disease	2 (3.7%)
Nodal Response	
Complete Response (CR)	51 (91%)
Partial Response (PR)	5 (9%)
Recurrence Patterns	
Local recurrence	7 (12.5%)
Regional recurrence	6 (10.7%)
Distant metastasis	15 (27.3%)
Median Time To Recurrence, Months (Range)	
Local	15 (12.06-17.25)
Regional	16 (9.6-24.7)
Distant	15.5 (4.6-29)
Late Toxicities	
Grade ≥3 GI toxicity	2 (3.6%)
Grade ≥3 GU toxicity	1 (1.8%)
Vaginal stenosis (all grades)	14 (25%)
Vaginal stenosis (Grade 3)	2 (3.6%)

Survival outcomes

Overall Survival

**Figure 1:**
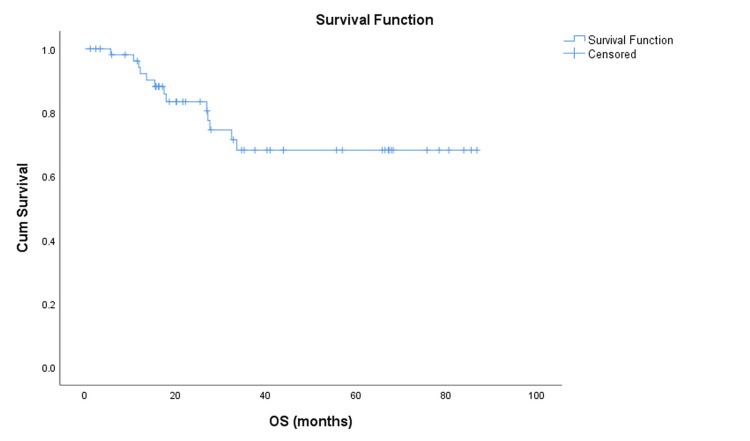
Kaplan–Meier curve showing overall survival (OS) Kaplan–Meier estimate of overall survival (OS) for the entire cohort (N = 56). At a median follow-up of 35 months, 13 patients (23.2%) had died, and 43 (76.8%) were censored. The mean OS was 65.9 months (95% CI: 56.3–75.4). Median OS was not reached due to the high proportion of censored observations.

At a median follow-up of 35 months, 13 (23.2%) patients had died, and 43 (76.8%) were censored. The mean overall survival was 65.9 months (95% CI: 56.3-75.4), and the Kaplan-Meier five-year OS estimate was 68% (Figure 1). The median OS was not reached because of the high proportion of censored observations.

Distant Recurrence Free Survival

**Figure 2:**
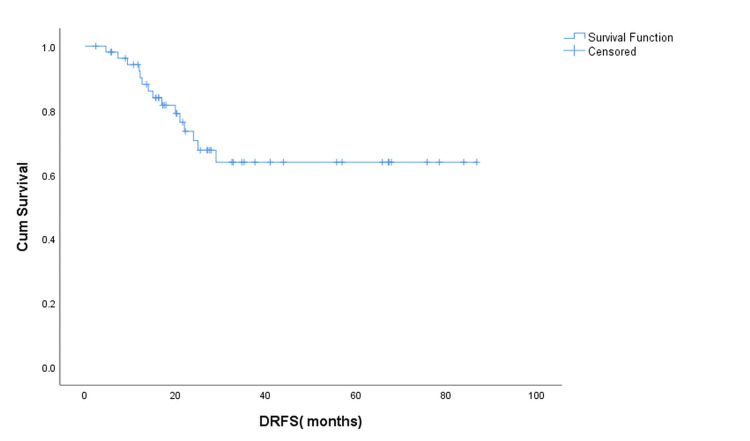
Kaplan–Meier curve showing distant recurrence-free survival (DRFS) Kaplan–Meier estimate of distant recurrence-free survival (DRFS) for the entire cohort (N = 56). At a median follow-up of 35 months, 16 patients (28.6%) developed distant recurrences, and 40 (71.4%) were censored. The mean DRFS was 60.8 months (95% CI: 50.4–71.1).

During follow-up, 16 (28.6%) patients developed distant recurrences, whereas 40 (71.4%) remained free of distant disease. The mean DRFS was 60.8 months (95% CI: 50.4-71.1), with a five-year DRFS estimate of 64% (Figure 2).

**Figure 3:**
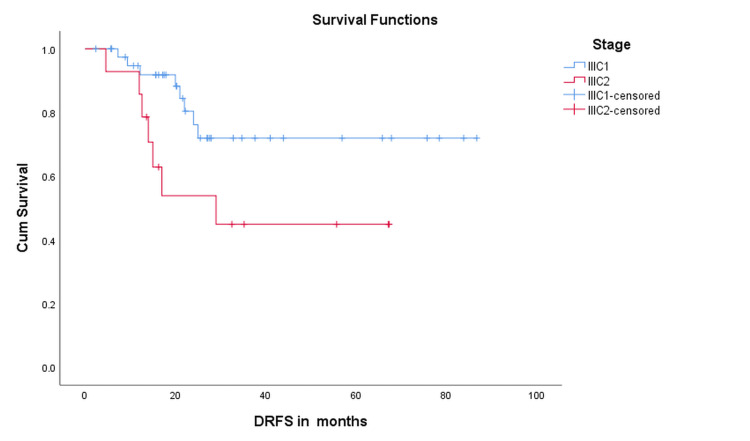
Kaplan–Meier curve comparing distant recurrence-free survival (DRFS) between FIGO stage IIIC1 and IIIC2 Kaplan–Meier comparison of DRFS between stage IIIC1 (N = 42) and stage IIIC2 (N = 14). The mean DRFS was 66.1 months (95% CI: 54.6–77.6) for IIIC1 versus 38.7 months (95% CI: 24.1–53.3) for IIIC2. Median DRFS for stage IIIC2 was 29 months (95% CI: 7.1–50.9), whereas it was not reached for stage IIIC1. A trend toward inferior DRFS was observed in stage IIIC2 compared with IIIC1 by the log-rank test (χ² = 3.404, df = 1, p = 0.065). CI: confidence interval; χ²: Chi-square statistic; df: degrees of freedom; p: p-value

In the subgroup analysis by stage, 9/42 (21.4%) of FIGO stage IIIC1 and 7/14 (50.0%) of stage IIIC2 developed distant recurrences. The mean DRFS was 66.1 months (95% CI: 54.6-77.6) for IIIC1 versus 38.7 months (95% CI: 24.1-53.3) for IIIC2. The median DRFS for stage IIIC2 was 29 months (95% CI: 7.1-50.9), whereas it was not reached for stage IIIC1 patients. Kaplan-Meier curves demonstrated a trend toward inferior DRFS in stage IIIC2 compared with IIIC1 (log-rank χ² = 3.404, p = 0.065; Figure 3). 

The subgroup analysis comparing stage IIIC1 and IIIC2 is presented in Table 3.

**Table 3:** Subgroup Analysis of DRFS by FIGO Stage Kaplan–Meier analysis comparing DRFS between FIGO stage IIIC1 and IIIC2 patients. Mean DRFS and median DRFS are shown with 95% CI. Survival distributions were compared using the log-rank test (χ² = 3.404, df = 1, p = 0.065). DRFS: distant recurrence-free survival; CI: confidence interval; χ²: Chi-square statistic; df: degrees of freedom; p: p-value

Stage	N of Events/Total	Mean DRFS (Months) (95% CI)	Median DRFS (Months) (95% CI)	p-value (Log-Rank)
IIIC1	9/42	66.1 (54.6–77.6)	Not reached	
IIIC2	7/14	38.7 (24.1–53.3)	29.0 (7.1–50.9)	p = 0.065

Regional Recurrence-Free Survival

**Figure 4:**
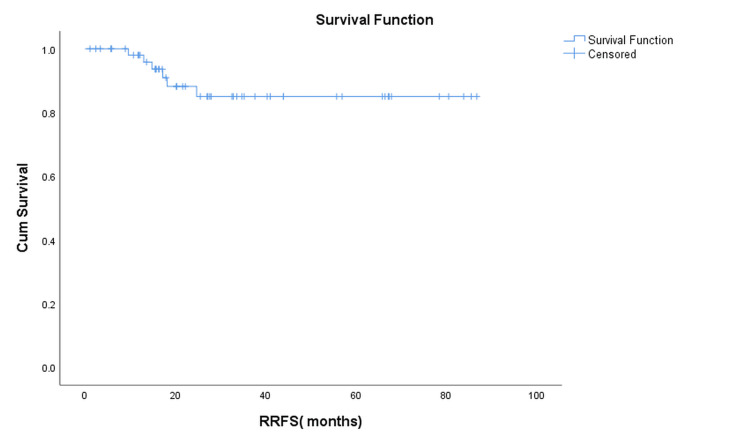
Kaplan–Meier curve showing regional recurrence-free survival (RRFS) Kaplan–Meier estimate of RRFS for the entire cohort (N = 56). At a median follow-up of 35 months, six patients (10.7%) developed regional nodal recurrence, and 50 (89.3%) remained free of relapse. The mean RRFS was 76.3 months (95% CI: 68.5–84.0).

Regional recurrence was observed in 6 (10.7%) patients, with a mean RRFS of 76.3 months (95% CI: 68.5-84.0). The median RRFS was not reached (Figure 4).

Local Recurrence-Free Survival

**Figure 5:**
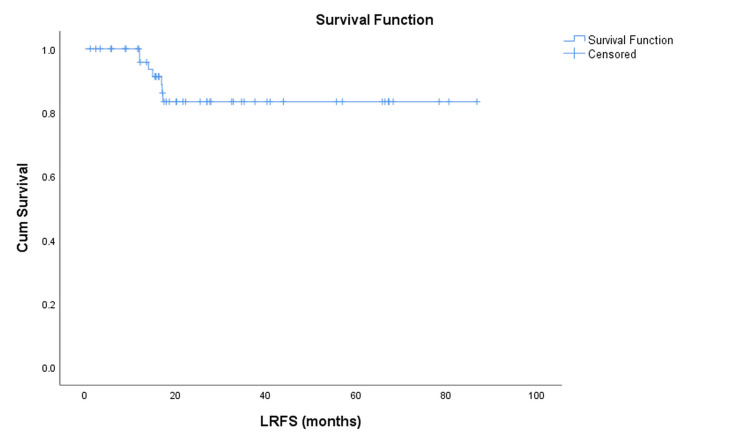
Kaplan–Meier curve showing local recurrence-free survival (LRFS) Kaplan–Meier estimate of LRFS for the entire cohort (N = 56). At a median follow-up of 35 months, seven patients (12.5%) developed local recurrence. The mean LRFS was 74.8 months (95% CI: 66.7–82.9), and the estimated 5-year LRFS rate was approximately 85–86%.

Local recurrence occurred in 7 (12.5%) patients. The estimated mean LRFS was 74.8 months (95% CI: 66.7-82.9 months). The three-year LRFS rate was approximately 88%, with an estimated five-year LRFS of 85-86% (Figure 5). All survival outcomes (OS, DRFS, RRFS, LRFS) are summarized in Table 4.

**Table 4:** Summary of Survival Outcomes (OS, DRFS, RRFS, and LRFS) Kaplan–Meier analysis was used to estimate survival outcomes. Mean and median survival times reported with 95% CI. No inter-group comparisons were performed in this table. CI: confidence interval

Outcome	Mean Survival (Months) (95% CI)	Median Survival (Months) (95% CI)	5-Year Survival (%)
Overall Survival (OS)	65.9 (56.3–75.4)	Not reached	68
Distant Recurrence-Free Survival (DRFS)	60.8 (50.4–71.1)	Not reached	64
Regional Recurrence-Free Survival (RRFS)	76.3 (68.5–84.0)	Not reached	≈85
Local Recurrence-Free Survival (LRFS)	74.8 (66.7–82.9)	Not reached	≈85–86

## Discussion

This single-institution retrospective study evaluated the outcomes of node-positive cervical cancer patients treated with definitive chemoradiation using IMRT/VMAT and SIB for radiologically involved lymph nodes. Our findings demonstrate high complete response rates at both the primary (46/56, 81.8%) and nodal sites (51/56, 91%), translating into encouraging five-year local and regional control rates of 86% and 85%, respectively, and an OS rate of 68%. Importantly, the treatment was well tolerated, with grade ≥3 gastrointestinal and genitourinary toxicities remaining below 5%.

The nodal response outcomes in this study are comparable to those of prior studies using SIB, which consistently reported complete response rates exceeding 85% [18,19]. Improved pelvic nodal control with doses > 55 Gy has been highlighted in earlier studies [13,14], and the durable regional control observed here aligns with those findings.

The survival outcomes were consistent with those reported in the published data. A large single-institution SIB dose-escalation study reported a five-year OS of 65% and pelvic control of 84% [17], which compares favorably with our five-year OS of 68% and local control of 86%. Similarly, extended-field IMRT boost studies have demonstrated regional control rates exceeding 90% [16], reinforcing the robustness of these findings. However, distant recurrence-free survival was only 64% at five years, emphasizing that systemic relapse remains the predominant mode of failure. Patients with stage IIIC2 disease experienced significantly inferior distant control compared with those with IIIC1 (median, 29 months vs. not reached; log-rank χ² = 3.404, p = 0.065), consistent with both Indian and international reports [21,22].

The toxicity profile of our cohort was favorable. Severe gastrointestinal and genitourinary late toxicities occurred in only 2 (3.6%) and 1 (1.8%) patients, respectively. Vaginal stenosis was reported in 14 patients (25%), with grade 3 in 2 patients (3.6%), consistent with the outcomes of the EMBRACE and RetroEMBRACE studies [24,25]. These findings align with those of previous reports that the IMRT/VMAT boost reduces bowel and bladder exposure compared with conventional approaches [15,20].

Collectively, these findings reinforce the feasibility and safety of IMRT/VMAT with nodal SIB for node-positive cervical cancer, particularly in the Indian setting, where prospective data are limited. By enabling conformal nodal dose escalation within OAR constraints, this strategy provides durable local and regional controls without compromising tolerability.

Future research should focus on prospective multi-institutional studies with larger patient cohorts to validate the outcomes of SIB in patients with node-positive cervical cancer. Incorporating functional imaging, such as PET-CT, for response-adapted nodal dose escalation and integrating novel systemic agents (including immunotherapy or targeted therapies) may improve distant control, especially in stage IIIC2. In addition, quality-of-life assessments and long-term toxicity monitoring should be included to better understand the balance between efficacy and tolerability of the treatment.

## Conclusions

This study demonstrates that definitive chemoradiation using IMRT/VMAT with an SIB to involved lymph nodes is both effective and well tolerated in patients with node-positive cervical cancer. High complete response rates and durable pelvic control were observed, with five-year local and regional control rates of 86% and 85%, respectively, and Overall Survival of 68%. Severe late gastrointestinal and genitourinary toxicities were uncommon, confirming the safety of this approach in routine practice. Despite these favorable local-regional outcomes, distant relapse emerged as the most common pattern of failure, especially among patients with para-aortic nodal disease who experienced poorer distant control compared with those with pelvic-only nodal involvement. These findings indicate that nodal dose escalation using modern radiotherapy techniques can be safely incorporated into clinical care, while also highlighting the need for systemic treatment intensification or novel strategies to reduce distant metastases in high-risk patients. Overall, this work supports the use of nodal SIB as a practical and achievable treatment strategy and provides a reference point for future prospective research.
